# Homogenization of locally resonant acoustic metamaterials towards an emergent enriched continuum

**DOI:** 10.1007/s00466-015-1254-y

**Published:** 2016-02-08

**Authors:** A. Sridhar, V. G. Kouznetsova, M. G. D. Geers

**Affiliations:** TU Eindhoven, Eindhoven, The Netherlands

**Keywords:** Locally resonant acoustic metamaterial, Computational homogenization, Model order reduction, Craig–Bampton Mode synthesis, Enriched continuum, Micro-inertia

## Abstract

This contribution presents a novel homogenization technique for modeling heterogeneous materials with micro-inertia effects such as locally resonant acoustic metamaterials. Linear elastodynamics is used to model the micro and macro scale problems and an extended first order Computational Homogenization framework is used to establish the coupling. Craig Bampton Mode Synthesis is then applied to solve and eliminate the microscale problem, resulting in a compact closed form description of the microdynamics that accurately captures the Local Resonance phenomena. The resulting equations represent an enriched continuum in which additional kinematic degrees of freedom emerge to account for Local Resonance effects which would otherwise be absent in a classical continuum. Such an approach retains the accuracy and robustness offered by a standard Computational Homogenization implementation, whereby the problem and the computational time are reduced to the on-line solution of one scale only.

## Introduction

The study of the elastodynamics of heterogeneous materials has led to the discovery of a special class of a composite material known as Acoustic Metamaterials [1]. They exhibit remarkable acoustic properties ranging from near zero transmissibility [2], enhanced absorption [3], negative dynamic mass density [4]/ bulk modulus [5], negative refractive index [6], super anisotropy, zero rigidity [7] etc. These exotic phenomena have numerous potential applications such as low frequency noise attenuation [3], isolation of civil structures from seismic waves [8], superlenses with a resolution beyond the Rayleigh limit [6, 9], waveguides that can be used to channel acoustic waves, etc.

Two important physical phenomena are responsible for the extraordinary properties of Acoustic Metamaterials, Local Resonance and Bragg Scattering [10]. These two phenomena operate at different length scales, whereby Bragg scattering is dominant for wavelengths (of the propagating wave) of the same order as the size of microstructural phases and Local Resonance at larger wavelengths. This work exclusively deals with the modeling of the latter phenomena, which are typically applied in the lower frequency regime. The subclass of Acoustic Metamaterials exhibiting Local Resonance is known as locally resonant acoustic metamaterials (LRAM). A typical unit cell microstructure of a LRAM consists of a matrix with an embedded inclusion or a substructure [10]. These inclusions/substructures consist of two parts, a central region with high mass density supported by a surrounding highly compliant region (for eg. rubber coated lead inclusions [2], also see Fig. 4). This enables the unit cell to exhibit low frequency localized vibration modes (see Fig. 5) that strongly couple to the long wavelength propagating wave in the matrix at the resonance frequency. The strong coupling around this frequency is what is responsible for the Local Resonance phenomena [2, 4].

A plethora of techniques is available for modeling the elastodynamics of heterogeneous materials although not all are equally suitable for describing LRAM. Direct numerical simulation (DNS) of LRAM structures using finite element method (FEM) is highly unpractical due to the large scale difference involved in such problems. Therefore it is necessary to develop efficient methods with more ingenuity. The Bloch-Floquet (or simply Bloch) theory [10] provides a general solution for the propagation of free waves (i.e. steady state waves in an infinite medium) in any periodic heterogeneous material. Substitution of the Bloch solution into the governing equations reduces the entire analysis to a single unit cell. The unit cell problem can then be solved numerically using various discretization methods such as Plane Wave Expansion [11], Variational Method [12], Finite Difference Time Domain [1] and FEM [13]. FEM provides a high flexibility in the design of the unit cell topologies at the cost of convergence with respect to the mesh size. This limitation can be overcome to a great extent by using model reduction techniques [14]. A homogenization scheme based on the Bloch theory was proposed by Willis [15, 16] and revisited again in [17]. The techniques based on Bloch theory has been highly successful in the study of free wave propagation but is mostly limited to this case. It is difficult if not impossible to account for complex transient loading and macroscopic boundary effects using this theory. Apart from Bloch theory, another generalized solution for transmission of free waves in a heterogeneous medium is given by the Multiple Scattering Theory [18]. It can be used to predict macroscopic transmission spectra and provides superior convergence with respect to discretization size, yet it is highly restricted to simple unit cell topologies. To the best of our knowledge it has only been implemented for spherical inclusions and granular media.

Another modeling approach is provided by Enriched or Micromorphic Continuum Theory [19], first proposed for elastodynamics by Mindlin [20]. It introduces additional macroscopic kinematic fields that account for the internal microscale dynamics in an otherwise homogeneous macroscopic medium. A new material property called ‘micro-inertia’ is postulated that characterizes the microdynamics. However, the only notable attempt at developing an enriched model that is especially capable of accounting for Local Resonance has been made by Sun et al. [21]. Homogenization, namely Asymptotic Homogenization [22–28] has also been a highly successful approach for modeling dispersion behavior in heterogeneous materials but few works exist that are specifically suitable for modeling LRAM.

The techniques developed thus far have been successful in studying LRAM behavior but do not provide a generalized description. A more comprehensive modeling technique for LRAM should enable the description and analysis of complex microstructure topologies, transient response at both scales and finite macroscopic structures with various boundary conditions. Motivated by this challenge, this contribution presents a novel framework for modeling LRAM that is not only general in its description but also highly efficient, enabling a fast and straightforward numerical implementation.

An extended first order Computational Homogenization framework [29] is taken as the point of departure to setup the multiscale transient dynamic problem. Except for linear elasticity and relaxed scale separation assumption the framework is general and the full balance of the linear momentum is solved at both scales.The relaxed scale separation principle introduced here retains the long wavelength (quasistatic) assumption on the matrix material but relaxes it on the inclusions. This accounts for the transient dynamic behavior of the microstructure characterizing micro-inertia effects, especially Local Resonance. A Computational Homogenization framework can be efficiently combined with FEM techniques for multiscale problems, which in turn gives the freedom to incorporate complex microsctructure topologies and finite macrostructure geometries, arbitrary transient excitation and sophisticated boundary conditions. The first use of Computational Homogenization to model LRAMs was made by Pham et al. [30]. The approach proposed in this work is distinctly different as it aims at obtaining a *closed-form* description of the macroscopic continuum, which is enriched to incorporate the effect of microscale dynamics. To eliminate the on-line (expensive) solutions of the microscale problems at each time step, typically used in a computational homogenization approach, a technique called the Craig Bampton Mode Synthesis [31] is employed. It entails a decomposition of the solution into two parts, the quasistatic response and internal dynamics, which makes it possible to condense the behavior of the microscale model upto the macroscopic level by applying homogenization. The method employs eigenmodes to extract the relevant dynamics of the microscale problem, which effectively captures Local Resonance effects with a minimum set of degrees of freedom. A highly compact closed-form description of a linear elastic LRAM results and the corresponding expressions effectively represent an enriched continuum where the emerging additional field accounts for the microscopic Local Resonance phenomena. This approach therefore provides a simple, efficient and general description of a linear elastic LRAM. This not only enables an efficient numerical implementation but also provides an intuitive understanding of the behavior of such materials.

The formulation used here to setup the micro and macroscale problems is defined for a Cauchy continuum in a two-dimensional space. The method can be easily extended to three-dimensions or other specialized continua such as shells, beams etc. The paper is organized as follows. Section 2 presents the overall methodology, including the homogenization framework for the problem and the techniques used to reduce the model towards an enriched continuum. In Sect. 3, the model is numerically validated against DNS for a 1D compressional wave test on a well known example of a LRAM structure. The conclusions are given in Sect. 4.

The following notations are used throughout the paper to represent different quantities and operations. The Cartesian basis vectors are given by $\mathbf {e}_k,\;k=1,2,3$. Unless otherwise stated scalars, vectors, second- and fourth-order Cartesian tensors are generally denoted by *a* (or *A*), $\mathbf {a}$, $\mathbf {A}$ and ${\mathbb {A}}$ respectively. The standard tensor operations are denoted as follows, dyadic product: $\mathbf {a}\otimes \mathbf {b}=a_ib_j\mathbf {e}_i\otimes \mathbf {e}_j$ , dot product: $\mathbf {A}.\mathbf {b}=A_{ij}b_j\mathbf {e}_i$ and double contraction: $\mathbf {A}:\mathbf {B}=A_{ij}B_{ji}$ (Einstein summation is used here and for all tensor operations). The conjugate of any second order tensor, $\mathbf {A}$ is indicated as $\mathbf {A}^\text { C}=A_{kj}\;{\mathbf {e}_j}\otimes \mathbf {e}_k$. Matrices of any type of quantity are in general denoted by $(\underline{\bullet })$ and the special case of a column matrix is denoted by . Submatrices of matrix $\underline{a}$ or  are denoted by the left superscript  and  respectively. Transpose of a matrix is given as $(\bullet )^\text { T}$.

## Homogenization and reduction methodology: towards an enriched continuum

The classical first order homogenization framework extended to the transient dynamic case is used to setup the multiscale problem. The full balance of linear momentum is considered at both scales. Scale transition relations are formulated that dictate the coupling between the scales. Once the framework is defined, the focus is shifted to the microscale problem which is reformulated in a discretized form. Using the Craig Bampton technique, a compact reduced model of the microstructure is obtained. The scale transition relations are then applied to upscale the reduced microscale balance equations, yielding the governing macroscale continuum equations, providing a closed form description of the enriched macroscopic continuum.

### Homogenization framework

#### Separation of scales

In the classical first order homogenization scheme, the separation of scales principle requires the following relation to hold true for a heterogeneous material with *n* microstructural constituents (cavities, grains, inclusions, matrix etc.):

1 $$\begin{aligned} l_j<<\lambda _{j}\,,\;\;j=1,\ldots ,n, \end{aligned}$$

where $l_j$ is the typical size of the *jth* microstructure constituent and $\lambda _{j}$ the corresponding shortest characteristic wavelength of the microstructural constituent for a given applied excitation. Under this assumption, the micro inertial response of the microstructure becomes negligible leading to a purely quasistatic response. Therefore a more relaxed scale separation principle is adopted here. Let $n_{het}$ and $n_{mat}$ be the number of microstructural phases constituting the heterogeneities and the core matrix, respectively:

2 $$\begin{aligned}&\text {Core matrix (long wavelength}\nonumber \\&\quad \text {approximation): }l_j<<\lambda _{j}^{mat} \,,\;\;&j=1,\ldots ,n_{mat}\,,\nonumber \\&\text {Heterogeneities: }{l_k\le \lambda _\text { k}^{het}} \,,\;\;&k=1,\ldots ,n_{het}\;, \end{aligned}$$

where $\lambda _j^{mat}$ and $\lambda _k^{het}$ are the shortest characteristic wavelengths in the *jth* and *kth* constituents of the matrix and the heterogeneity for a given applied excitation, respectively. The long wavelength approximation still applies to the matrix whereas a more relaxed hypothesis holds for the heterogeneities. The microstructural lengths can now scale with the wavelengths associated to heterogeneities, incorporating possible micro-inertia effects.

#### Macroscale problem

Let ${\mathcal {D}}_\text { M}$ represent the domain of the macroscale problem and $\partial {\mathcal {D}}_\text { M}$ its boundary. Let $\mathbf {x}_\text { M}$ give the position vector of any point in this domain. A Cauchy continuum is assumed with the displacement vector $\mathbf {u}_\text { M}$ and its gradient $\varvec{\nabla }_\text { M}\mathbf {u}_\text { M}$ representing the primary kinematic fields. It will be shown later that static rotational equilibrium is not necessarily *a priori* satisfied at each continuum point which requires the use of the full displacement gradient instead of the symmetric linear strain tensor. The governing equation in the absence of external body forces is given by the linear balance of momentum,

3 $$\begin{aligned} \varvec{\nabla }\,.\,\varvec{\sigma }_\text { M}-\dot{\mathbf {p}}_\text { M}=\mathbf {0}, \end{aligned}$$

where $\varvec{\sigma }_\text { M}$ and $\mathbf {p}_\text { M}$ are the macroscopic stress tensor and momentum respectively. Appropriate initial and boundary conditions should be applied but these are not explicitly stated here since they are not important for the subsequent derivations. The macroscale constitutive response^1^ is derived from the solution of the microscopic problem via homogenization. In classical Computational Homogenization schemes, this macroscopic constitutive response is not obtained through a closed-form equation. This is different (and better) for the present case, see Sect. 2.3, which constitutes an important step forward.

#### Microscale problem

To each material point of ${\mathcal {D}}_\text { M}$, a fine scale domain ${\mathcal {D}}$ with boundary $\partial {\mathcal {D}}$ is associated, where the microscale problem is defined. The domain is selected such that it captures the local microstructural effects at that point. It is termed the representative volume element (RVE). For (locally) periodic microstructures, the RVE is defined as the unit cell that spans the (local) microstructure. For the sake of simplicity, no specific subscripts are used to indicate the variables associated to the microstructural domain. Let the total volume and the infinitesimal volume element of ${\mathcal {D}}$ be represented by *V* and $\mathrm {d}V$ respectively and an infinitesimal surface element of $\partial {\mathcal {D}}$ be represented by $\mathrm {d}S$. A Cauchy continuum is assumed for the microscopic problem with the kinematic field variables given by displacement $\mathbf {u}$ and linear strain $\varvec{\epsilon }$. In order to capture the micro inertial effects, the full balance of momentum has to be considered in the RVE,

4 $$\begin{aligned} \varvec{\nabla }\cdot \varvec{\sigma }-\dot{\mathbf {p}}=\mathbf {0}. \end{aligned}$$

A linear elastic material is considered. For every material constituent domain , the following constitutive relations hold, 5a5b Where $\varvec{\nabla }^\text { sym}\mathbf {u}=\frac{1}{2}(\varvec{\nabla }\mathbf {u}+(\varvec{\nabla }\mathbf {u})^\text { C})$ and  and  stand for the mass density and the elastic material stiffness of the constituent $\alpha$ respectively. A perfect bonding condition is assumed; 6a6b Here  are the unit normal vectors to the interface between both constituents. The initial and boundary conditions on the RVE required to solve the micro problem will be defined upon introducing the scale transition relations.

#### General microscopic kinematics

The kinematics of the RVE corresponding to a point $\mathbf {x}_\text { M}$ of ${\mathcal {D}}_\text { M}$ is given by the following first order representation of the microscopic kinematics at that point,

7 $$\begin{aligned} \mathbf {u}=\mathbf {u}_\text { M}+(\varvec{\nabla }_\text { M}\mathbf {u}_\text { M})^\text { C} \,\cdot \,(\mathbf {x}-\mathbf {x}_\text { R})+\mathbf {w}. \end{aligned}$$

Here, $\mathbf {x}_\text { R}$ is a reference vector whose definition will be given later and $\mathbf {w}$, called the microfluctuation field, represents the fine scale variations due to the microstructure heterogeneities. This field provides the necessary kinematic degrees of freedom to describe the Local Resonance phenomena.

#### Scale transition relations

With the introduction of the microfluctuation field $\mathbf {w}$, additional constraints in form of boundary conditions on the RVE are required to ensure the well posedness of the problem. These conditions usually follow from the (chosen) relations coupling both scales. They essentially represent the kinematic coupling between the macro and microscale and are called downscaling relations. In the micro-macro direction upscaling relations recover the macroscopic stress and momentum from the solution of the RVE boundary value problem. They result by inserting the downscaling relations into the Hill-Mandel macrohomogeneity condition, generalized to the transient dynamic case. The up and downscaling relations together constitute the scale transition relations that dictate the coupling between the two scales.

*Downscaling relations (kinematic boundary conditions)*: The condition on the macroscopic displacement is formulated as the overall rigid body displacement of the RVE. This is equivalent to constraining the microfluctuation at a single arbitrary point, say , on the RVE boundary to zero.8The second condition on the macroscopic displacement gradient follows the established averaging theorem [32]

9 $$\begin{aligned} \frac{1}{V}\underset{{\mathcal {D}}}{\int }\varvec{\nabla }\mathbf {u}\,\mathrm {d}V =\varvec{\nabla }_\text { M}\mathbf {u}_\text { M}. \end{aligned}$$

Substituting the RVE kinematics given by Eq. (7) into the above expression and simplifying results in

10 $$\begin{aligned} \int _{{\mathcal {D}}}\varvec{\nabla }\mathbf {w}\mathrm {d}V=\varvec{0}. \end{aligned}$$

**Fig. 1:**
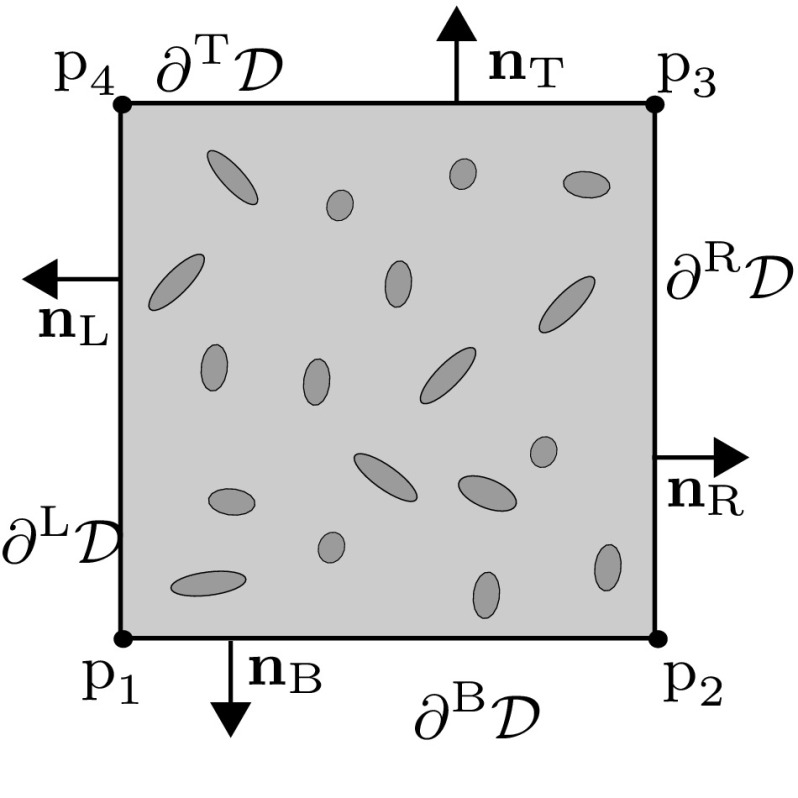
Sketch of the boundaries of a RVE and its normal vectors

This gives the constraints on the microfluctuation field, which is easily converted to a boundary integral by applying Gauss theorem

11 $$\begin{aligned} \int _{\partial {\mathcal {D}}}\mathbf {n}\otimes \mathbf {w}\mathrm {d}S=\varvec{0}, \end{aligned}$$

where $\mathbf {n}$ is the outward normal to the RVE boundary. Relation (11) gives the minimal kinematic constraint that enforces Eq. (9) for a given macroscopic displacement gradient. It has been shown in the literature that this constraint is too weak and generally leads to poor results for small RVEs. Therefore a stronger form known as periodic boundary condition (pbc) is applied instead, which provides much better RVE size convergence [33]. The assumption of periodic boundary displacements can be justified for a transient problem if the matrix is assumed to behave quasi-statically. Indeed, this holds true in the case of the relaxed scale separation principle given by Eq. (2), which retains the long wavelength assumption on the matrix. The periodic boundary conditions can be formulated as follows. Considering a RVE with a periodic rectangular shape (in 2D), the boundary is split into four edges: left, right, bottom and top, denoted by L, R, B and T, respectively. The four vertices are denoted by $\text {p}_1$, $\text {p}_2$, $\text {p}_3$ and $\text {p}_4$ (see Fig. 1). Let the position vectors of the vertices be represented by , ,  and , respectively. The normal unit vector at every corresponding pair of points on opposite boundary sides satisfies,  and . By constraining the microfluctuations on the edges to be periodic, i.e. 12a12b

Equation (11) is automatically satisfied. Making the choice,  in Eq. (8) and substituting it in Eq. (12), gives the following condition on the microfluctuations at nodes p$_1$, p$_2$ and p$_4$ (which will be used in a later section)13*Upscaling relations* (*Hill-Mandel condition*): According to the principle of virtual work, the total internal virtual work must be conserved for a dynamic system for any imposed kinematics. The Hill-Mandel principle extends this concept to relate the two scales where the volume averaged virtual work of the RVE is equated to its macroscopic equivalent in a material point. Therefore, equating the volume average of the virtual work of Eqs. (3) and (4) gives

14 $$\begin{aligned}&\frac{1}{V}\underset{{\mathcal {D}}}{\int }\varvec{\sigma }:\delta (\varvec{\nabla }^\text { sym}\mathbf {u}) \mathrm {d}V\,+\,\frac{1}{V}\underset{{\mathcal {D}}}{\int }{\dot{\mathbf {p}}}\,\cdot \,\delta \mathbf {u}\mathrm {d}V\nonumber \\&\quad =\varvec{\sigma }_\text { M}:\delta (\varvec{\nabla }_\text { M} \mathbf {u}_\text { M})\,+ \;{\dot{\mathbf {p}}}_\text { M}\,\cdot \,\delta \mathbf {u}_\text { M}. \end{aligned}$$

Rewriting the left hand side of the above expression in terms of the virtual work of the external boundary tractions $\mathbf {t}=\mathbf {n}.\varvec{\sigma }$ on the RVE gives,

15 $$\begin{aligned} \frac{1}{V}\underset{\partial {\mathcal {D}}}{\int }\mathbf {t} \cdot \delta \mathbf {u}\,\mathrm {d}S=\varvec{\sigma }_\text { M}: \delta (\varvec{\nabla }_\text { M}\mathbf {u}_\text { M})+ \dot{\mathbf {p}}_\text { M}\cdot \delta \mathbf {u}_\text { M}. \end{aligned}$$

Substituting Eq. (7) into the above expression and making use of Eqs. (12) and (13) gives

16 $$ \begin{aligned}&\frac{1}{V}\underset{\partial {\mathcal {D}}}{\int }\mathbf {t}\,\cdot \,\delta \mathbf {u}_\text { M}\,\mathrm {d}S\!+\!\frac{1}{V}\underset{\partial {\mathcal {D}}}{\int }\mathbf {t}.\delta (\varvec{\nabla }_\text { M}\mathbf {u}_\text { M})^\text { C}\cdot (\mathbf {x}-\mathbf {x}_\text { R})\,\mathrm {d}S\!+\!\frac{1}{V}\underbrace{\underset{\partial {\mathcal {D}}}{\int }\mathbf {t}\cdot \delta \mathbf {w}\,\mathrm {d}S}_{=0,\;\text {via (8)}\, \& \,\text {(12)}}\nonumber \\&\quad =\varvec{\sigma }_\text { M}: \delta (\varvec{\nabla }_\text { M}\mathbf {u}_\text { M})+ \dot{\mathbf {p}}_\text { M}\,\cdot \,\delta \mathbf {u}_\text { M}. \end{aligned}$$

Which upon simplification reads,

17 $$\begin{aligned} \varvec{\sigma }_\text { M}&=\frac{1}{V}\underset{\partial {\mathcal {D}}}{\int } \mathbf {t}\otimes (\mathbf {x}-\mathbf {x}_\text { R})\,\mathrm {d}S, \end{aligned}$$

18 $$\begin{aligned} \dot{{\mathbf {p}}}_\text { M}&=\frac{1}{V}\underset{\partial {\mathcal {D}}}{\int }\mathbf {t}\,\mathrm {d}S. \end{aligned}$$

This gives the corresponding upscaling relations. Note that in homogenization of static problems, $\mathbf {x}_\text { R}$ vanishes from Eq. (17) due to static equilibrium (zero net traction on the boundary) of the RVE. This is no longer the case in a dynamic setting where the net tractions do not vanish, see Eq. (18), indicating the dependency of the macroscopic stress on $\mathbf {x}_\text { R}$ and also on the micro-inertial effects. By employing Gauss theorem and the microscopic balance given by Eq. (4), the boundary integral form of Eqs. (17) and (18) can be written in the equivalent volume integral form as follows,

19 $$\begin{aligned} \varvec{\sigma }_\text { M}&=\frac{1}{V}\underset{{\mathcal {D}}}{\int }(\varvec{\sigma } +\dot{\mathbf {p}}\otimes (\mathbf {x}-\mathbf {x}_\text { R}))\,\mathrm {d}V, \end{aligned}$$

20 $$\begin{aligned} \mathbf {p }_\text { M}&=\frac{1}{V}\underset{{\mathcal {D}}}{\int }\mathbf {p}\,\mathrm {d}V. \end{aligned}$$

Note that Eq. (19) highlights the explicit coupling of the macroscopic stress to the microscopic momentum, again indicating the influence of micro-inertial effects on the macroscopic stress.

### Discretization and model reduction

Using standard discretization techniques, e.g. based on the finite element method (FEM), the discrete balance of momentum of the RVE can be written as follows21Here  and  represent the nodal displacements, accelerations and applied forces respectively in which the quantities associated to each node are represented by a vector and assembled in a vector column fashion. $\underline{\mathbf {K}}$ represents the stiffness tensor matrix and $\underline{\mathbf {M}}$ the mass tensor^2^ matrix which transform the nodal displacement and acceleration vectors respectively into internal nodal force vectors. In the derivations that ensue, standard Galerkin projection is used to perform model reduction.

#### Periodic boundary condition

In order to apply the periodic boundary conditions it is necessary to partition the system into tied (constrained) and retained nodes denoted by the characters ‘t’ and ‘r’ respectively. The tied nodes constitute the right and the top edge nodes along with vector point $\text {p}_3$ and the retained nodes constitute the remaining boundary nodes and the nodes in the RVE interior. The discrete form of Eq. (12) can be written in terms of displacements as follows,222324where  denotes a column with unit scalar entries of the same size as  and . The above expressions give the constraint relations on the tied nodes in terms of the retained nodes. Let $\underline{\mathop {{T}}\limits ^{{^\text { *}}}}$ represent the scalar reduction matrix reflecting this linear transformation;25Applying the reduction (25) on Eq. (21) leads to the reduced discrete governing equations in terms of the retained nodes26where27

#### Craig Bampton reduction

The Craig Bampton Mode Synthesis [31] (or Craig Bampton for/CBMS short) is a popular substructuring technique used in structural dynamics to obtain reduced models of complex assembled systems (e.g. cars, planes etc). It involves a reduced description of the dynamic response of the interior of every subsystem or substructure with respect to prescribed displacements at the external boundary of each subsystem. The total response is then obtained by assembling the individual reduced substructure models at the boundaries separating the substructures. This concept can be applied to a multiscale framework where the microscale problem is treated as a substructure of the macroscale problem. Eigenmodes of the interior dynamics are used as a reduced basis, which perfectly capture the Local Resonance phenomena.

**Fig. 2:**
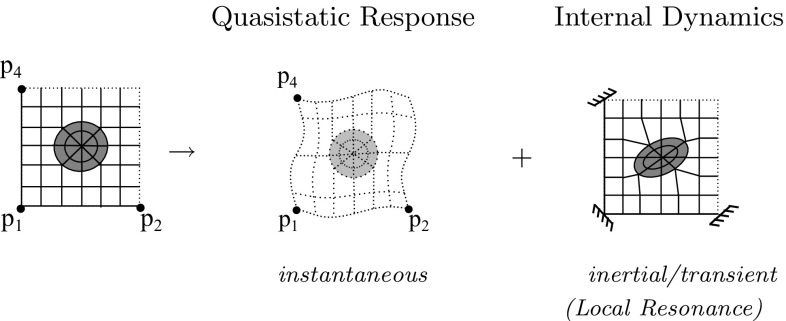
Illustration of the Craig Bampton decomposition. The total dynamic response of an RVE to prescribed boundary displacements can be represented as a superposition of its quasistatic response and its internal dynamics spanned by a set of eigenmodes with the prescribed nodes fixed

With the periodic boundary conditions incorporated, the Craig Bampton procedure can now be applied. The total dynamic response of any structure under prescribed displacements (velocities, accelerations) can be expressed as a superposition of its corresponding quasistatic response and its internal dynamics spanned by a minimal set of eigenmodes of the structure with the prescribed nodes fixed. This is illustrated in Fig. 2. This decomposition is essential since it separates the Local Resonance effects from the rest of the mechanical response thereby enabling the construction of a reduced model. The quasistatic response gives the instantaneous mechanical response whereas the internal dynamics represents the inertial response due to Local Resonance. The two subproblems are next treated independently and later superposed to obtain the total solution. Note that the incorporation of internal dynamics is made possible due to the assumption of the relaxed scale separation principle given by Eq. (2). In general, when applied incorrectly, the Craig Bampton reduction could lead to stiff and sometimes incorrect responses since the eigenmodes are computed on a constrained system, which may not capture the actual dynamics. However this is not an issue for homogenization as long as the prescribed displacement nodes lie on the matrix (which does not exhibit relevant dynamical behavior in the frequency ranges satisfying the relaxed separation of scales) and not the inclusion.

In order to proceed with the derivations, the retained nodes must be further partitioned into prescribed and free nodes denoted by ‘p’ and ‘f’, respectively. The prescribed nodes include nodes p$_1$, p$_2$ and p$_4$ and the free nodes consist of the remaining nodes. The partitioned form of Eq. (26) is written as28where  represents a column of length $N_\text {f}$ (where $N_\text {f}$ indicates the number of free nodes) in which each entry is a zero vector. It can be seen that no external forces act on the free nodes.

*Quasistatic Response*: The quasistatic response is recovered by omitting the mass contribution in Eq. (28), enabling to solve for the free degrees of freedom using a static condensation procedure. The solution can then be represented in terms of the prescribed displacements as follows (subscript ‘qs’ is used for quasistatic)29The term $\underline{\mathbf {S}}$ represents the static condensate (also known as the Schur complement) of the stiffness matrix $\underline{\mathop {\mathbf {K}}\limits ^{{\text { *}}}}$, and  represents a $3\times 3$ matrix with each entry containing a second order identity tensor. The expression for $\underline{\mathbf {S}}$ reads30Using Eq. (29) as a reduced basis, model reduction of Equation (28) gives the governing equations for the quasistatic response expressed in displacements of the prescribed nodes only31where,32Here, the superscript $(\bullet )^\text { CT}$ represents the transpose of a matrix in which each of the tensor entries are conjugated.

*Internal dynamics*: The internal dynamics is added to the solution in order to compensate for neglecting the contribution of the inertial forces when calculating the quasistatic response. This correction is essential in accounting for Local Resonance. The solution of the dynamic subproblem is expressed as a superposition of $N_\text {q}$ eigenmodes (here $N_\text {q}<<2N_\text {f}$, $2N_\text {f}$ being the number of free degrees of freedom), computed with prescribed nodes fixed. The expression for this reads33where $\underline{{\varvec{\varPhi }}}$ represents the vector matrix containing the eigenmodes,  the corresponding column of generalized scalar displacements, i.e. the amplitude of the eigenmodes also referred to as modal displacements and  represents a $3\times N_\text {q}$ zero vector matrix. The eigenvalue problem and the mass normalization condition of each eigenmode , where $s=1,2,\ldots ,N_\text {q}$ are respectively, given as3435where  is the eigenfrequency corresponding to  and  represents a zero vector column of length $N_\text {f}$. To yield a computationally efficient model, the basis should be constructed such that it only contains the essential (or excited) modes that trigger Local Resonance. A more quantitative mode selection criteria is briefly described in Sect. 2.3. In addition, the retained eigenmodes must also satisfy the scale separation principle given by Eq. (2). Applying Eq. (33) to reduce Eq. (21) with the help of Eqs. (34) and (35) gives36where  is a zero scalar column of length $N_\text {q}$ and,37Note that Eq. (36) describes a set of $N_\text {q}$ uncoupled spring mass systems with eigenfrequencies given by $\underline{\omega }$. This gives the simplest description of Local Resonance without any coupling to the macroscopic dynamics. This coupling will be obtained by combining the solutions of the two subproblems.

*Linear superposition*: The full solution is now expressed as a superposition of the quasistatic response  and the internal dynamics . Combining Eqs. (29) and (33) gives,38Projecting Eq. (28) on the subspace of Eq. (38) gives the reduced coupled dynamic model of the RVE.3940where41is the vector matrix providing the coupling between the two equations.

### Emerging enriched continuum

The discrete form of the downscaling relations given by Eq. (13) with account for Eq. (7) is represented as42where  is an identity column of length 3 and , where  is a unit scalar column of size 3. Similarly, the discrete form of the upscaling relations Eqs. (17) and (18) is given as4344The macroscopic constitutive relations can now be recovered by substituting Eqs. (40) and (42) into the above expressions:4546Here $\mathbf {O}$ stands for the zero tensor and $(\bullet )^\text { LC}$ stands for the left conjugate of a higher order tensor defined as $A_{jikl}^\text { LC}=A_{ijkl}$. As shown in the above expression, several terms are zero or can be neglected. The corresponding justification for each of these terms is given below:

$$\mathbf {O}^{(\text {i})}$$:
These terms are identically zero because  is in fact the null space of $\underline{{\mathbf {K}}}_\text {qs}$ as it describes the rigid body modes imposed by the prescribed nodes.
$$\mathbf {O}^{(\text {ii})}$$:
This term gives the elastic inertia (inertial contribution to deformation modes) and for the materials of concern here it is negligible compared to the elastic stiffness (i.e. ). Although the total term,  can become significant at higher applied frequencies, $\omega$ (since $\varvec{\nabla }_\text { M}\ddot{\mathbf {u}}_\text { M} \propto \omega ^2$), these frequencies approach the homogenizability limit (where scale separation is violated), hence it is justified to drop this term in the considered regime.
$$\mathbf {O}^{(\text {iii})}$$:
These are the cross coupling terms between $\varvec{\sigma }_\text { M}$ and $\ddot{\mathbf {u}}_\text { M}$ and $\dot{\mathbf {p}}_\text { M}$ and $\varvec{\nabla }_\text { M}\ddot{\mathbf {u}}_\text { M}$. They are in general not zero and depend on the choice of $\mathbf {x}_\text { R}$, which was yet unspecified. The choice of $\mathbf {x}_\text { R}$ does not influence the accuracy of the final solution. Hence, the value of $\mathbf {x}_\text { R}$ is simply chosen such that these terms become zero. The physical interpretation of these terms will be unraveled in future work.

**Fig. 3:**
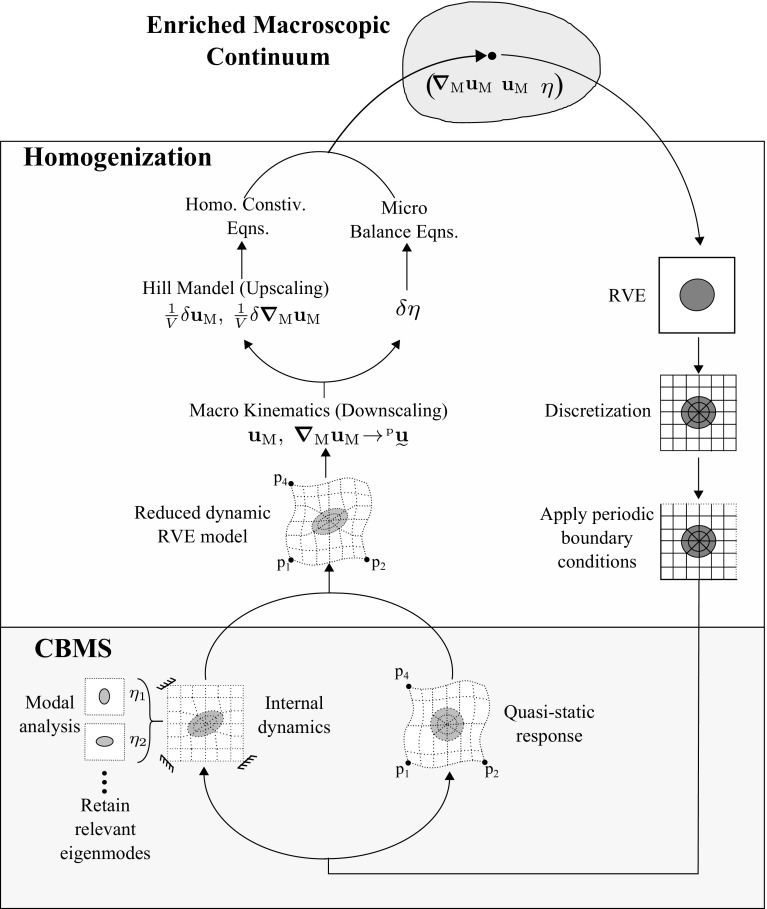
Summary of the main steps involved in computing the enriched continuum model

The homogenized constitutive expressions given by Eqs. (45) and (46) can now be written in a compact closed-form as follows,4748where, 49a49b49c49d Here ${\mathbb {C}}_\text { M}$ represents the effective elasticity tensor, $\rho _\text { M}$ is the average mass density of the RVE. The terms  and  represent the coupling of the *sth* modal degree of freedom to the macroscopic stress and momentum respectively. They can be used to determine the relevant eigenmodes for the reduced basis. Local resonance modes must have at least one nonzero (and finite) coefficient in ${\mathbf {H}}$ or ${\mathbf {j}}$. The displacement gradient in Eq. (47) can be replaced with the linear strain tensor due to the symmetries present in the homogenized elasticity tensor ${\mathbb {C}}_\text { M}$. However  can be asymmetric since rotational eigenmodes can exist, introducing angular inertia into the system violating the rotational equilibrium condition accompanying the symmetry of the stress tensor. Hence, this triggers the asymmetry of the macroscopic stress tensor in materials with micro-inertia, in general. In the example problem considered in this work, the rotational modes have a negligible Local Resonance amplitude and thus the effect of angular inertia does not contribute here. They will be discussed in more detail in forthcoming work.

Finally, the balance of momentum of the internal dynamics needs to be expressed in its homogenized continuum notations. Applying Eqs. (42) to (39) gives50The emerging macroscopic governing equations of the enriched continuum can be summarized below

Macroscopic balance of momentum.

51 $$\begin{aligned} \varvec{\nabla }.\varvec{\sigma }_\text { M}-\dot{\mathbf {p}}_\text { M}=\mathbf {0}. \end{aligned}$$

*Microscopic balance of momentum. (To be solved at the macroscale)*52Homogenized constitutive relations. 53a53b

Note that ${\eta }$ is an emergent field variable, which effectively ‘enriches’ the macroscopic continuum with the micro-inertia effects, in the micromorphic sense as initially defined by Eringen [19]. It constitutes an internal field variable that does not explicitly appear in the classical macroscopic balance equation, and is therefore appropriately termed as internal dynamics.

The entire procedure can be summarized as follows. Starting from the discretized balance of momentum of the RVE, periodic boundary conditions are first applied to the boundary nodes. The Craig Bampton decomposition is introduced by expressing the solution as the superposition of the quasistatic response and the internal dynamics represented by an optimum eigenmode basis. This expression is then used to obtain a reduced balance of momentum of the system. The scale transition relations are then applied to transform the reduced balance of momentum into the homogenized enriched macroscopic continuum equations. The eigenmode problem is typically solved off-line, in advance. The macroscale on-line solution procedure therefore reduces to a single scale enriched problem. The procedure is illustrated in Fig. 3.

## Numerical validation

### Local resonance acoustic metamaterial (LRAM)

**Fig. 4:**
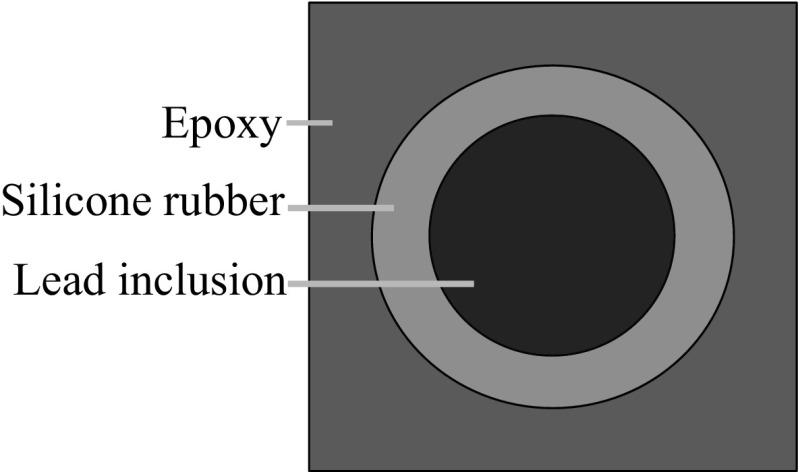
Example of a LRAM unit cell designed by [2]

For the purpose of validation of the presented model, a well known and understood LRAM design of [2] is adopted. The structure of the unit cell and the macroscopic lattice structure are shown in Fig. 4. It consists of an epoxy matrix with an embedded lead inclusion coated with soft rubber. In [2] spherical inclusions were used. Since derivations are carried out in 2D space, the inclusions are modeled as infinite cylinders instead. The geometric and material properties of the LRAM are given in Table 1. The high compliance of rubber combined with the high mass density of lead compared to the epoxy triggers the observed low frequency localized resonance modes.

**Table 1:** The geometric and material properties of the unit cell under study

Geometrical properties		Material properties			
Radius of lead inclusion	5 mm	Parameter	Lead	Rubber	Epoxy
Thickness of rubber coating	2.5 mm	$\rho \; (\times 10^3\;\; \text {kg/m}^3)$	11.6	1.3	1.18
Length of the unit cell	20.1 mm	$\lambda$ ($\text {N/m}^2$)	$4.23\times 10^{10}$	$6\times 10^5$	$4.43\times 10^{9}$
		$\mu$ ($\text {N/m}^2$)	$1.49\times 10^{10}$	$4\times 10^4$	$1.59\times 10^9$

**Fig. 5:**
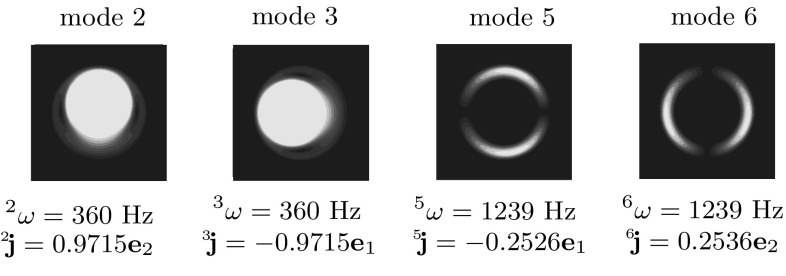
Local resonance mode shapes and their associated eigenfrequencies and momentum coupling coefficients of the unit cell under consideration

The FEM model of the RVE was constructed using the Marc Mentat software package. The discretized system contains close to 6000 degrees of freedom. An eigenvalue analysis was carried out on the system and the first 10 in plane eigenmodes were extracted. Out of these 10 modes, only 2, 3, 5 an 6 are Local Resonance modes with finite values of the momentum coupling coefficient. The remaining modes have either zero or negligible values of their respective stress and momentum coupling coefficients. Modes 2 and 3 and 5 and 6 are degenerate with eigenfrequencies 360 Hz and 1239 Hz respectively. The mode shapes, the eigenfrequencies and the momentum coupling coefficients of each mode are given in Fig. 5. Modes 3 and 5 are coupled along the $\mathbf {e}_1$ direction and modes 2 and 6 are coupled along the $\mathbf {e}_2$ direction. The mode shapes overlap strongly with those found in [2], hence it can be concluded that these are indeed the Local Resonance modes.

A simple estimation can be made to verify if the RVE under consideration satisfies the scale separation criteria. By neglecting the Local Resonance effects, an approximate estimation of the matrix resonance frequency reads

54 $$\begin{aligned} \omega _\text { mat}\approx \frac{c_\text { Mij}}{2L}, \end{aligned}$$

where *L* is the length of the RVE and $c_\text { Mij}=\sqrt{\frac{C_\text { Mij}}{\rho _\text { M}}}$ is the speed of sound for a given wave mode ($c_\text { M11}$ indicates compressive wave and $c_\text { M12}$, shear wave, etc). The above expression gives the order of the frequency at which the matrix starts behaving dynamically and the relaxed scale separation is violated. Therefore, it can be used to obtain an estimation of the limiting frequency. Substituting the values of the respective quantities into the above expression gives a value of the order of $10^4$ Hz for both shear and compressive wave modes. The frequencies of interest determined by the Local Resonance eigenfrequencies (360 and 1239 Hz) lie well within the limiting frequency. Thus the LRAM under consideration is well suited for applying the proposed homogenization method.

**Fig. 6:**
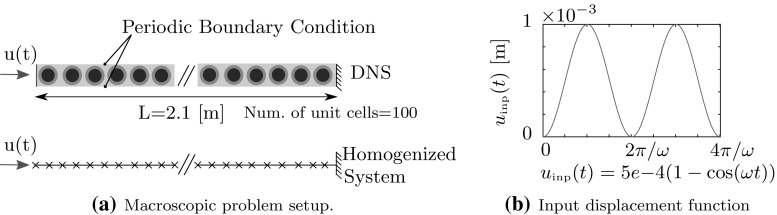
Construction of the macroscopic compression wave test using DNS and the enriched homogenized model. The right edge is constrained and a prescribed displacement $u_\text { inp}(t)$ is applied on the left edge. **a** Macroscopic problem setup. **b** Input displacement function

### Macroscopic problem construction

The developed homogenized enriched continuum is next verified against DNS. For the sake of simplicity, the macroscopic problem is restricted to a 1D compressional wave propagation test. The DNS model is constructed by sequentially stacking 100 RVEs as shown in Fig. 6a. In order to ensure an effective 1D macroscopic behavior for this 2D system, periodic boundary conditions are applied to the top and bottom edges of the RVEs to mimic an infinitely large structure in the vertical direction. The right edge of the system is fully clamped, and prescribed displacements are applied on the left edge. A harmonic loading function of frequency $\omega$ is applied on the left end for 2 time periods as shown in Fig. 6b. The finite element model of the total DNS system contains over 500,000 degrees of freedom.

The homogenized model of the system is constructed by discretizing a 1D enriched continuum using 100 linear finite elements with 101 nodes. The problem is discretized in time using an implicit Newmark scheme. The nodal degrees of freedom consist of the horizontal macroscopic displacement and the two generalized modal displacements associated with Mode 3 and 5 (only the modes providing coupling in $\mathbf {e}_1$ direction are retained since the problem considered here only involves wave propagation in this direction). The right most node is constrained and the prescribed displacements are applied on the left most node (see Fig. 6a).

**Fig. 7:**
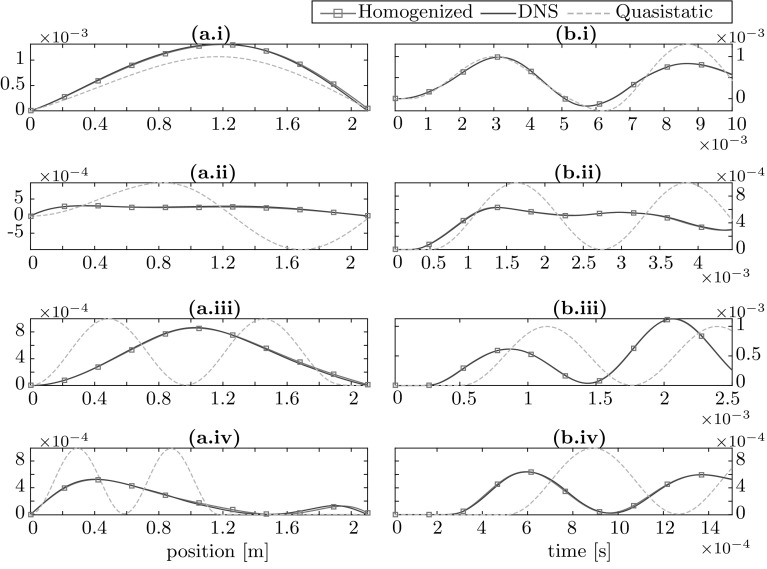
Plot of horizontal nodal displacement [m] versus **a** position after two time periods of the input excitation ($t=4\pi /\omega$) and **b** time at position x $=$ 0.42 m for excitation frequency (*i*) $\omega =200$ Hz, (*ii*) $\omega =450$ Hz, (*iii*) $\omega =800$ Hz and (*iv*) $\omega =1330$ Hz

Four transient simulations tests were performed at different excitation frequencies $\omega$, which are selected to probe various regions of interest. It is well known in the literature [2], that the Local Resonance coupling is strongest in the frequency regions above each eigenfrequency known as ‘stop bands’. For the simulation, the first excitation frequency is selected at 200 Hz which is far below the first eigenfrequency where the Local Resonance effects should have a weak influence. The response is expected to be predominantly quasistatic at this frequency. The second frequency is selected at 450 Hz which is in the first stop band. The Local Resonance coupling will be strong there. The third frequency is selected at 800 Hz which is in between the two stop bands and the fourth is selected at 1330 Hz in the second stop band. The results are shown in Fig. 7. Two plots of the horizontal displacement are displayed for each excitation frequency, one as a function of position after two time periods and the second as a function of time at x $=0.42$ m. The homogenized solution matches the DNS results in an excellent manner, thus validating the developed method.

A quasistatic homogenization solution (i.e. the simulation with Local Resonance effects ignored) is also shown in order to be able to judge the impact of the Local Resonance coupling. At 200 Hz, the behavior of the LRAM is still captured reasonably well by the quasistatic solution. At 400 Hz, the quasistatic solution cannot reproduce the LRAM behavior at all, showing the pronounced influence of the Local Resonance phenomenon which is picked up remarkably well by the proposed homogenization scheme. Even though the transient simulation is carried out for only 2 time periods without damping, a strong wave decay effect of the stop band is clearly visible in the simulation. At 800 Hz, propagating waves reappear in the LRAM but the wavelength and speed are significantly higher compared to that of the quasistatic wave. This is due to the fact that the resonance of the first eigenmode causes decoupling of the mass from its host matrix reducing the overall effective dynamic mass density of the system. This in turn causes the wave speed to increase resulting in a longer wavelength. Finally, at 1330 Hz, the wave decay phenomena is again active, this time due to the second stop band.

To conclude, the above results illustrate the importance of correctly accounting for the micro-inertia effects in homogenizing the behavior of LRAM. The proposed enriched continuum resulting from the homogeniation and reduction scheme is effective in achieving the correct response.

## Conclusions

This paper presented a novel approach towards the modeling and analysis of LRAM with linear elastic constituents. By applying the Craig Bampton reduction to a transient dynamic Computational Homogenization framework, a compact closed form description has been obtained. The resulting equations reveal that an enriched continuum model emerged, with additional degrees of freedom representing the internal dynamics of the microstructure. The method was validated against the DNS solution for a 1D transient compressional wave test of a LRAM structure consisting of a rubber coated lead inclusions at different excitation frequencies. An excellent match was thereby obtained. An order of 3 decade reduction in the problem size with respect to DNS was achieved without any loss of accuracy. The comparison of the results with the quasistatic solution showed the strong influence of Local Resonance on the macroscopic dynamics beyond the low frequency (long wavelength) quasistatic regime.

The main features and advantages of the developed technique, that distinguishes it from other available techniques for the modeling and analysis of materials with Local Resonances (or long wavelength micro-inertia effects in general), can be summarized as follows,

- *Generality*: The full balance of momentum is considered at both scales, which allows the incorporation of complex boundary conditions, transient loading and arbitrary topologies.
- *Efficiency*:
  - Model reduction allows for a highly compact representation of the microscale problem which naturally implies improved numerical efficiency.
  - An Enriched macroscopic homogenized continuum description emerges, allowing to use any appropriate solution technique. Therefore discretization schemes with superior convergence properties such as Spectral Element Method etc. can be used as well.
  - It also offers an additional benefit over standard transient dynamic multiscale implementations in the fact that the homogenization procedure has to only be carried out once and not at each time step.
- *Extendability*: Many possibilities exist for further extending the present formulation, e.g. to incorporate moderate nonlinearities [34, 35], damping, multiphysical effects, material nonlocality (second order gradient) etc.

In conclusion, the approach presented here aims to pave the path towards integrating core capabilities of Computational Mechanics into the realm of Acoustic Metamaterials. This could provide a breakthrough needed for the practical design and production of such materials for real world applications.

## Footnotes

1 Note that the constitutive model includes both the relation for the macroscopic stress and momentum in terms of the kinematic variables.

2 Since scalar value shape functions are usually used to discretize the system, the mass matrix would in general be a scalar matrix, but for convenience of derivations, it has been transformed to a tensor matrix by multiplication with an identity tensor.

## References

[1] Hussein MI, Leamy MJ, Ruzzene M (2014). Dynamics of phononic materials and structures: historical origins, recent progress, and future outlook. Appl Mech Rev.

[2] Liu Z, Zhang X, Mao Y, Zhu YY, Yang Z, Chan CT, Sheng P (2000). Locally resonant sonic materials. Science.

[3] Wen J, Zhao H, Lv L, Yuan B, Wang G, Wen X (2011). Effects of locally resonant modes on underwater sound absorption in viscoelastic materials. J Acoust Soc Am.

[4] Sheng P, Mei J, Liu Z, Wen W (2007). Dynamic mass density and acoustic metamaterials. Phys B.

[5] Ding Y, Liu Z, Qiu C, Shi J (2007). Metamaterial with simultaneously negative bulk modulus and mass density. Phys Rev Lett.

[6] Zhu R, Liu XN, Hu GK, Sun CT, Huang GL (2014). Negative refraction of elastic waves at the deep-subwavelength scale in a single-phase metamaterial. Nat Commun.

[7] Lai Y, Wu Y, Sheng P, Zhang Z (2011). Hybrid elastic solids. Nat Mater.

[8] Mitchell SJ, Pandolfi A, Ortiz M (2014). Metaconcrete: designed aggregates to enhance dynamic performance. J Mech Phys Solids.

[9] Pendry JB (2000). Negative refraction makes a perfect lens. Phys Rev Lett.

[10] Deymier PA (2013). Acoustic metamaterials and phononic crystals.

[11] Sigalas M, Economou EN (1993). Band structure of elastic waves in two dimensional systems. Solid State Commun.

[12] Goffaux C, Dehesa JS (2003). Two-dimensional phononic crystals studied using a variational method: application to lattices of locally resonant materials. Phys Rev B.

[13] Farzbod F, Leamy MJ (2011). Analysis of bloch’s method and the propagation technique in periodic structures. J Vib Acoust.

[14] Krattiger D, Hussein MI (2014) Bloch mode synthesis: Ultrafast methodology for elastic band structure calculations. Phys Rev E 90(6):063306 10.1103/PhysRevE.90.063306 25615221

[15] Willis JR (2012). The construction of effective relations for waves in a composite. Comptes Rendus Mécanique.

[16] Milton GW, Willis JR (2007). On modifications of newton’s second law and linear continuum elastodynamics. Proc R Soc.

[17] Nassar H, He QC, Auffray N (2015). Willis elastodynamic homogenization theory revisited for periodic media. J Mech Phys Solids.

[18] KafesakI M (1999). Multiple-scattering theory for three-dimensional periodic acoustic composites. Phys Rev B.

[19] Eringen AC (1999). Microcontinuum field theories.

[20] Mindlin RD (1964). Microstructure in linear elasticity. Arch Ration Mech Anal.

[21] Zhu R, Huang HH, Huang GL, Sun CT (2011). Microstructure continuum modeling of an elastic metamaterial. Int J Eng Sci.

[22] Wang Z, Sun CT (2002). Modeling micro-inertia in heterogeneous materials under dynamic loading. Wave Motion.

[23] Andrianov IV, Bolshakov VI, Danishevs VV, Weichert D (2008). Higher order asymptotic homogenization and wave propagation in periodic composite materials. Proc Roy Soc.

[24] Craster RV, Kaplunov J, Pichugin AV (2010). High-frequency homogenization for periodic media. Proc Roy Soc.

[25] Chen W, Fish J (2000). A dispersive model for wave propagation in periodic heterogeneous media based on homogenization with multiple spatial and temporal scales. J Appl Mech.

[26] Bacigalupo A, Gambarotta L (2014). Second-gradient homogenized model for wave propagation in heterogeneous periodic media. Int J Solids Struct.

[27] Boutin C, Rallu A, Hans S (2014). Large scale modulation of high frequency waves in periodic elastic composites. J Mech Phys Solids.

[28] Chesnais C, Boutin C, Hans S (2012). Effects of the local resonance on the wave propagation in periodic frame structures: generalized Newtonian mechanics. J Acoust Soc Am.

[29] Geers MGD, Kouznetsova VG, Brekelmans WAM (2008). Multi-scale computational homogenization: trends and challenges. J Comput Appl Math.

[30] Pham K, Kouznetsova VG, Geers MGD (2013). Transient computational homogenization for heterogeneous materials under dynamic excitation. J Mech Phys Solids.

[31] Craig RR, Bampton MCC (1968). Coupling of substructures for dynamic analyses. Am Inst Aeronaut Astronaut.

[32] Nemat-Nasser S, Hori M (1993). Micromechanics: overall properties of heterogeneous materials.

[33] Terada K, Hori M, Kyoya T, Kikuchi N (2000). Simulation of the multi-scale convergence in computational homogenization approaches. Int J Solids Struct.

[34] Marion M, Temam R (1989). Nonlinear galerkin methods. SIAM J Numer Anal.

[35] Wu L, Tiso P (2015). Nonlinear model order reduction for flexible multibody dynamics: a modal derivatives approach. Multibody Syst Dynam.

